# A new Miocene baleen whale from the Peruvian desert

**DOI:** 10.1098/rsos.160542

**Published:** 2016-10-05

**Authors:** Felix G. Marx, Naoki Kohno

**Affiliations:** 1 Department of Geology and Palaeontology, National Museum of Nature and Science, Tsukuba, Japan; 2 School of Biological Sciences, Monash University, 25 Rainforest Walk, Clayton, Victoria 3800, Australia; 3 Geosciences, Museum Victoria, Melbourne, Australia; 4 Directorate of Earth and History of Life, Royal Belgian Institute of Natural Sciences, Brussels, Belgium; 5 Graduate School of Life and Environmental Sciences, University of Tsukuba, Tsukuba, Japan

**Keywords:** Mysticeti, baleen whale, rorqual, Balaenopteridae, lunge feeding, Pisco Formation

## Abstract

The Pisco-Ica and Sacaco basins of southern Peru are renowned for their abundance of exceptionally preserved fossil cetaceans, several of which retain traces of soft tissue and occasionally even stomach contents. Previous work has mostly focused on odontocetes, with baleen whales currently being restricted to just three described taxa. Here, we report a new Late Miocene rorqual (family Balaenopteridae), *Incakujira anillodefuego* gen. et sp. nov., based on two exceptionally preserved specimens from the Pisco Formation exposed at Aguada de Lomas, Sacaco Basin, southern Peru. *Incakujira* overall closely resembles modern balaenopterids, but stands out for its unusually gracile ascending process of the maxilla, as well as a markedly twisted postglenoid process of the squamosal. The latter likely impeded lateral (omega) rotation of the mandible, in stark contrast with the highly flexible craniomandibular joint of extant lunge-feeding rorquals. Overall, *Incakujira* expands the still meagre Miocene record of balaenopterids and reveals a previously underappreciated degree of complexity in the evolution of their iconic lunge-feeding strategy.

## 1. Introduction

Balaenopterids include the largest animals on the Earth, and represent more than half of all extant species of baleen whales (Mysticeti) [1]. Unusually for a large mammal, new species of rorquals continue to be discovered [2–4], further emphasizing their dominance in the modern whale fauna. Balaenopterids stand out for their highly distinctive lunge-feeding strategy, which involves the engulfment of vast amounts of water and prey in an expandable throat pouch, followed by water expulsion and baleen-assisted filtering [5–7]. The mechanics of this behaviour—arguably one of the most extreme shown by any mammal—are becoming increasingly better understood [7–12], but far less is known about how and when balaenopterid lunge feeding first arose.

Divergence dating places the initial diversification of extant balaenopterids—here taken to include the closely related grey whale, *Eschrichtius robustus*—in the Middle or Early Miocene, with proposed dates ranging from 20 to 13 Ma [13–17]. Nevertheless, the Miocene history of rorquals remains poorly known, and is currently restricted to just five Late Miocene species from Italy (*Plesiobalaenoptera quarantellii*) [18], Peru (*Balaenoptera siberi*) [19,20] and the West Coast of the United States (‘*Balaenoptera*’ *ryani*, ‘*Megaptera*’ *miocaena*, *Parabalaenoptera baulinensis*) [21–23]. Additional Late or latest Miocene records, e.g. from Japan [24,25], Italy [26], South Africa [27] and California [28], are fragmentary and/or in need of further study.

The western coast of South America has particular promise for expanding the Miocene record of balaenopterids, because of both the abundance and the generally high quality of preservation of cetacean fossils from this region [29–33]. Here, one of the richest sources is the Miocene–Pliocene Pisco Formation exposed in the coastal Pisco-Ica and Sacaco Basins of southern Peru [30,31,34]. Previous work has focused mostly on odontocetes, which are represented by a diverse Miocene assemblage including physeteroids, ziphiids, pontoporiids, phocoenids and kentriodontids [30,31,35,36]. By contrast, and despite their local abundance [31,36], only three mysticetes from the Pisco Formation have so far been reliably named: the cetotheriid *Piscobalaena nana* [37,38]; the neobalaenine *Miocaperea pulchra* [39]; and the balaenopterid ‘*Balaenoptera*’ *siberi* [19,20]. The status of the enigmatic *Piscocetus sacaco*, known only from a single fragmentary and apparently privately owned specimen [40], needs to be clarified. Here, we describe a new genus and species of Late Miocene rorqual based on two exceptionally preserved specimens from Aguada de Lomas (Sacaco Basin), and discuss the implications of our new taxon for balaenopterid phylogeny and feeding ecology.

## 2. Material and methods

The two specimens described here were collected, exported and prepared by Siber & Siber (Switzerland) in 1989–1990, with permission of the Peruvian authorities. The material is now housed at the Gamagori Natural History Museum, Gamagori and the Kanagawa Prefectural Museum of Natural History, Odawara, both located in Japan. Additional preparation of GNMH Fs-098-12, including the removal of the left bulla, was carried out locally in Gamagori using a pneumatic air scribe. Morphological nomenclature and tympanoperiotic orientation follow Mead & Fordyce [41], unless indicated. Measurements of both specimens are reported in tables 1–4. We included the new material into the total evidence matrix of Marx *et al*. [42], and ran a non-clock Bayesian analysis (four chains, three runs, temp. = 0.1, 50 million generations) in MrBayes v. 3.2.6 [43], on the Cyberinfrastructure for Phylogenetic Research (CIPRES) Science Gateway [44]. In addition, we performed a second analysis with the same settings, but based on the morphological data only. For both analyses, convergence was judged based on the average standard deviation of split frequencies (total evidence: 0.012; morphology only: less than 0.01) and results were summarized with the first 25% of generations discarded as burn-in. This published work and the nomenclatural acts it contains have been registered in ZooBank. The LSID for this publication is: urn:lsid:zoobank.org:pub:DF696255-5BD3-435C-B3C3-EE39F9EB33F3.

**Table 1.** Measurements (in millimetres) of the holotype (GNHM Fs-098-12) and paratype (KPM NNV730) skulls of *Incakujira anillodefuego*.

	GNHM Fs-098-12	KPM NNV730
bizygomatic width	1000	844
condylobasal length	2260	1800 (estimated)
maximum width of temporal fossa	350	286
bicondylar width	250	215
anteroposterior diameter of orbit	175	176
maximum width of anterior portion of premaxilla	85	76
maximum width of ascending process of premaxilla	23	18 (right)
		24 (left)
width of ascending process of maxilla at base	25	25
width of ascending process of maxilla at tip	5	8
maximum length of nasal	190	196
width of nasal at anterior border	52	58
width of nasal at tip of narial process of frontal	28	28
maximum width of narial fossa	205	138

**Table 2.** Measurements (in millimetres) of the holotype (GNHM Fs-098-12) ear bones of *Incakujira anillodefuego*.

	GNHM Fs-098-12
tympanic bulla	
maximum length	89.5
width just anterior to sigmoid process	56.3
maximum height	50.5 (estimated)
width of sigmoid process	15.5
height of sigmoid process in lateral view (to base of sigmoid cleft)	35.4
malleus	
maximum dimension	15.6
maximum height of head	10.4
width of manubrium	7.8
length of anterior process (from base of mallear ridge)	17.4

**Table 3.** Measurements (in millimetres) of the holotype (GNHM Fs-098-12) and paratype (KPM NNV730) forelimbs of *Incakujira anillodefuego*.

	GNHM Fs-098-12	KPM NNV730
scapula, maximum length	780	585
scapula, maximum height	460	358 (estimated)
humerus, maximum proximodistal length including epiphyses	340	299
humerus, maximum width of shaft in lateral view	140	123 (estimated)
radius, maximum proximodistal length	510	428
ulna, maximum proximodistal length excluding olecranon	445	361

**Table 4.** Measurements (in millimetres) of the holotype (GNHM Fs-098-12) and paratype (KPM NNV730) vertebral columns of *Incakujira anillodefuego*. Lumbars and caudals of GNHM F2-098-12 are not included as their individual identifications cannot be determined with certainty.

	GNHM Fs-098-12	KPM NNV730
total body length, including the skull	8250	7150
cervical		
atlas	90	71
thoracic		
T1	75	—
T5	100	—
T6	100 (estimated)	—
T7	107	—
T8	120	—
T9	140	111
T10	140	124
T11	145	120
T12	135	122
T13		126
lumbar		
L1	—	130
L5	—	152
L6	—	158
L7	—	163
L8	—	169
L9	—	168
caudal		
Ca1	—	173 (L10?)
Ca2	—	168 (L11?)
Ca3	—	165 (Ca1?)
Ca4	—	160 (Ca2?)
Ca5	—	154 (Ca3?)
Ca6	—	147 (Ca4?)
Ca7	—	138 (Ca5?)
Ca8	—	125 (Ca6?)
Ca9	—	112 (Ca7?)
Ca10	—	80+ (Ca8?)
Ca11	—	65 (Ca9?)
Ca12	—	60 (Ca10?)
Ca13	—	56 (Ca11?)
Ca14	—	53 (Ca12?)
Ca15	—	49 (Ca13?)
Ca16	—	33+ (Ca14?)
Ca17	—	27 (Ca15?)
Ca18	—	21+ (Ca16?)

### 2.1. Institutional abbreviations

GNHM, Gamagori Natural History Museum, Gamagori, Japan; KMNH, Kitakyushu Museum of Natural and Human History, Kitakyushu, Kyushu, Japan; KPMNH, Kanagawa Prefectural Museum of Natural History, Odawara, Japan; MNHN, Museum National d'Histoire Naturelle, Paris, France; NMNS, National Museum of Nature and Science, Tsukuba, Japan.

## 3. Systematic palaeontology

Cetacea Brisson, 1762

Mysticeti Gray, 1864

Balaenopteridae Gray, 1864

*Incakujira* gen. nov.

*LSID.* urn:lsid:zoobank.org:act:AB0A1646-D7BC-4333-A2D2-F9D3F611B1C3

*Type species*. *Incakujira anillodefuego* sp. nov.

*Etymology*. Named after the Inca Empire that ruled pre-Columbian Peru. ‘Kujira’ is the Japanese term for ‘whale’.

*Diagnosis*. As for type and only species.

*Incakujira anillodefuego* sp. nov.

*LSID.* urn:lsid:zoobank.org:act:5BE0B9B5-AA86-4F54-B0C7-D8279044B064

*Holotype*. GNHM Fs-098-12, a nearly complete skeleton preserving mineralized baleen, but with an incomplete posterior portion of the tail. Casts of the tympanic bulla and the periotic are housed at the National Museum of Nature and Science in Tsukuba, Japan (NMNS PV23792).

*Paratype*. KPM NNV730, nearly complete, partially prepared skeleton.

**Figure 1.**
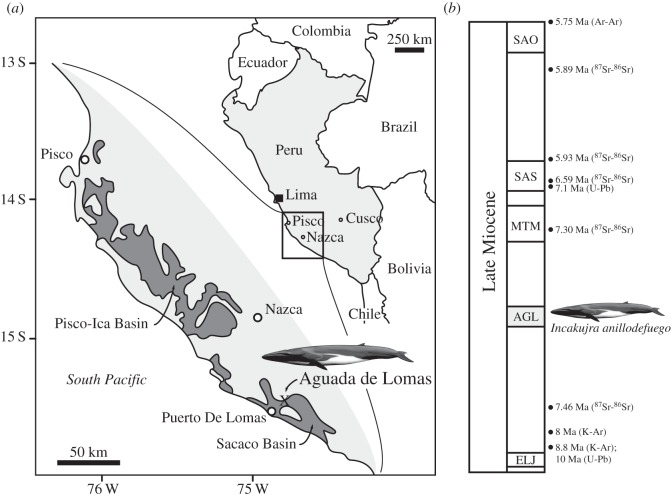
Type locality and horizon of *Incakujira anillodefuego*. (*a*) Location of Aguada de Lomas within the Sacaco Basin, southern Peru; (*b*) stratigraphic column of the Pisco Formation, modified from [45], with the horizon that yielded *I. anillodefuego* indicated on the right. Abbreviations mark the following vertebrate horizons: AGL, Aguada de Lomas; ELJ, El Jahuay; MTM, Montemar; SAS, Sud Sacaco (West); SAO, Sacaco. Drawing of balaenopterid by C. Buell.

*Locality and horizon.* Aguada de Lomas, near Puerto de Lomas, approximately 80 km south of Nazca, Peru (approximate coordinates S 15°30′34′′, W 74°47′57′′; figure 1). Both the holotype and the paratype come from *Cosmopolitodus*-bearing horizons of the Pisco Formation (H.-J. Siber 2015, personal communication), with KPM NNV730 specifically originating from the ‘upper *Isurus* [*Cosmopolitodus*] zone’, and thus most likely the AGL level [30,36] (C. de Muizon 2016, personal communication); 8–7 Ma based on K-Ar, Sr and zircon U-Pb dating, with a possible date range of 7.5–7.3 Ma based on strontium dating only [30,45].

*Etymology.* After the Pacific Ring of Fire, which connects Peru with Japan.

*Diagnosis.* Small balaenopterid sharing with other members of the family the presence of an abruptly depressed supraorbital process of the frontal, anteroposteriorly elongate ascending processes of the maxilla and premaxilla, an anteroposteriorly thickened postorbital ridge, a cranially elongated pars cochlearis of the periotic, a dorsoventrally narrow mandibular foramen located posterior to the level of the coronoid process, and a subcondylar furrow that extends around the posterior face of the mandible. Differs from all extinct and extant balaenopterids except *Eschrichtius* in having an ascending process of the premaxilla that is transversely wider than that of the maxilla, and from all balaenopterids except *Eschrichtius* and ‘*Balaenoptera*’ *portisi* in having a markedly twisted postglenoid process.

Within the context of the subfamily Eschrichtiinae, *I. anillodefuego* gen. et sp. nov. further differs from *Eschrichtius* and *Eschrichtioides* in lacking a U-shaped orbitotemporal crest, and in having a much narrower ascending process of the maxilla, an anteroposteriorly broader supraorbital process of the frontal, a well-defined pocket between the ascending process of the maxilla and the anteromedial corner of the supraorbital, a more anteriorly projected supraoccipital, and a well-developed anterolateral shelf on the tympanic bulla; from *Eschrichtius* and *Gricetoides* in having a fenestra rotunda that is separated from the aperture for the cochlear aqueduct; from *Eschrichtius* and ‘*Balaenoptera*’ *portisi* in having a transversely broader rostral portion of the maxilla, a broad overlap of the posterior portion of the ascending process of the maxilla with the anterior portion of the parietal, and a dorsoventrally thickened orbital rim of the supraorbital process (in lateral view); and from ‘*Balaenoptera*’ *portisi* in having a less attenuated, anteriorly truncated supraoccipital, a well-defined external occipital crest, and less exposure of the parietal on the vertex.

In terms of all other balaenopterids, *I. anillodefuego* gen. et sp. nov. differs from *Archaebalaenoptera* in having a less attenuated rostrum, an ascending process of the premaxilla that extends posteriorly as far as the ascending process of the maxilla and the nasal, a less concave lateral margin and a more distinctly truncated anterior margin of the supraoccipital, and a dorsoventrally thickened orbital rim of the supraorbital process; from ‘*Balaenoptera*’ *ryani* in having a cranially elongated pars cochlearis, a well-developed external occipital crest, a more clearly anteriorly truncated supraoccipital, relatively larger occipital condyles, and less exposure of the parietal on the vertex; from *Balaenoptera*, *Diunatans*, *Megaptera novaeangliae* and ‘*Megaptera*’ *hubachi* in having a more prominent, anteroposteriorly thickened paroccipital process; from *Balaenoptera*, *Diunatans*, *Fragilicetus* and ‘*Megaptera*’ *hubachi* in having a well-developed external occipital crest and a triangular protuberance on the supraoccipital; and from *Balaenoptera*, *Diunatans* and *M. novaeangliae* in having a much narrower, attenuated ascending process of the maxilla and a distally expanded compound posterior process of the tympanoperiotic.

Differs from *Balaenoptera* in having an anteriorly expanded premaxilla, a less anteroposteriorly expanded postorbital process, an anterior process of the malleus that is not fused to the dorsomedial corner of the sigmoid process of the tympanic bulla, and in lacking a squamosal crease; from *Fragilicetus* in in having a much narrower, attenuated ascending process of the maxilla, a straight or sigmoidal posterior border of the supraorbital process, and a squamosal that does not bulge into the temporal fossa; from ‘*Megaptera*’ *hubachi* in having a more elongate nasal, a well-developed narial process of the frontal, a more attenuated supraoccipital and a more cranially elongated pars cochlearis; and from *M. novaeangliae* in having a straight, rather than concave, anterior border of the supraorbital process, a relatively narrower narial fossa, and a less transversely expanded posterior portion of the cranium.

Differs from ‘*Balaenoptera*’ *siberi* and ‘*Megaptera*’ *miocaena* in having a much narrower, attenuated ascending process of the maxilla, a more attenuated supraoccipital, and an anteroposteriorly broader supraorbital process; from ‘*Balaenoptera*’ *siberi* in having the frontal and (inter)parietal exposed on the vertex; from ‘*Megaptera*’ *miocaena* in having a less anteriorly projected supraoccipital and a distally more expanded compound posterior process of the tympanoperiotic; from *Parabalaenoptera*, *Plesiobalaenoptera* and *Protororqualus* in having a somewhat more elongate and slender rostrum; from *Parabalaenoptera* and *Plesiobalaenoptera* in having a well-defined pocket between the ascending process of the maxilla and the anteromedial corner of the supraorbital; from *Parabalaenoptera* in having a more robust nasal, a proportionally larger occipital condyle that projects to or beyond the level of the paroccipital process, and a less transversely expanded transverse process of the axis with a relatively larger vertebrarterial foramen; from *Plesiobalaenoptera* in having a narrower ascending process of the maxilla, a less clearly defined antorbital process, and a distally expanded compound posterior process of the tympanoperiotic; and from *Protororqualus* in lacking a prominent postorbital process, and in having an anteriorly truncated supraoccipital, a postorbital process of the frontal that is closely juxtaposed to the zygomatic process of the squamosal, and an ascending process of the premaxilla that extends as far posteriorly as the ascending process of the maxilla and the nasal.

## 4. Description

### 4.1. Overview

**Figure 2.**
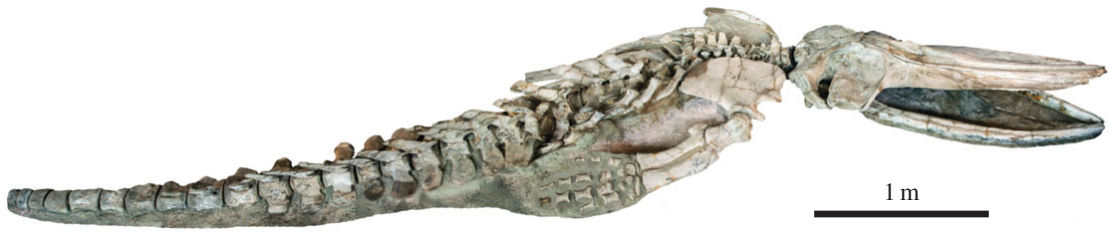
Holotype of *Incakujira anillodefuego*. Specimen GNHM Fs-098-12, as currently on display at the Gamagori Natural History Museum, Gamagori, Japan.

The holotype skeleton (GNHM Fs-098-12) is exceptionally well preserved, articulated and nearly complete (figure 2). The specimen was preserved dorsal side up. Notably absent are an unknown number of caudal and likely also lumbar vertebrae, several phalanges, the right lacrimal and jugal, the hyoid apparatus, both pelves and all chevron bones. The missing posterior vertebrae were likely lost as a result of surface exposure and erosion, as indicated by the much more weathered surface of the caudal vertebrae when compared with all other parts of the specimen. Except for the skull, most of the ventral surface of the skeleton has either not been prepared or is fixed in position for display. The skull is virtually intact and, for the most part, preserved in its original shape, despite abundant fractures caused by post-burial sediment compaction. The only major damage affects the anterior half of the rostrum and the lower jaws, which sediment pressure left both partially crushed and, in the case of the rostrum, flattened and somewhat bent dorsally. For display purposes, the skull has been propped open above the mandibles, so as to create the impression of an open mouth.

**Figure 3.**
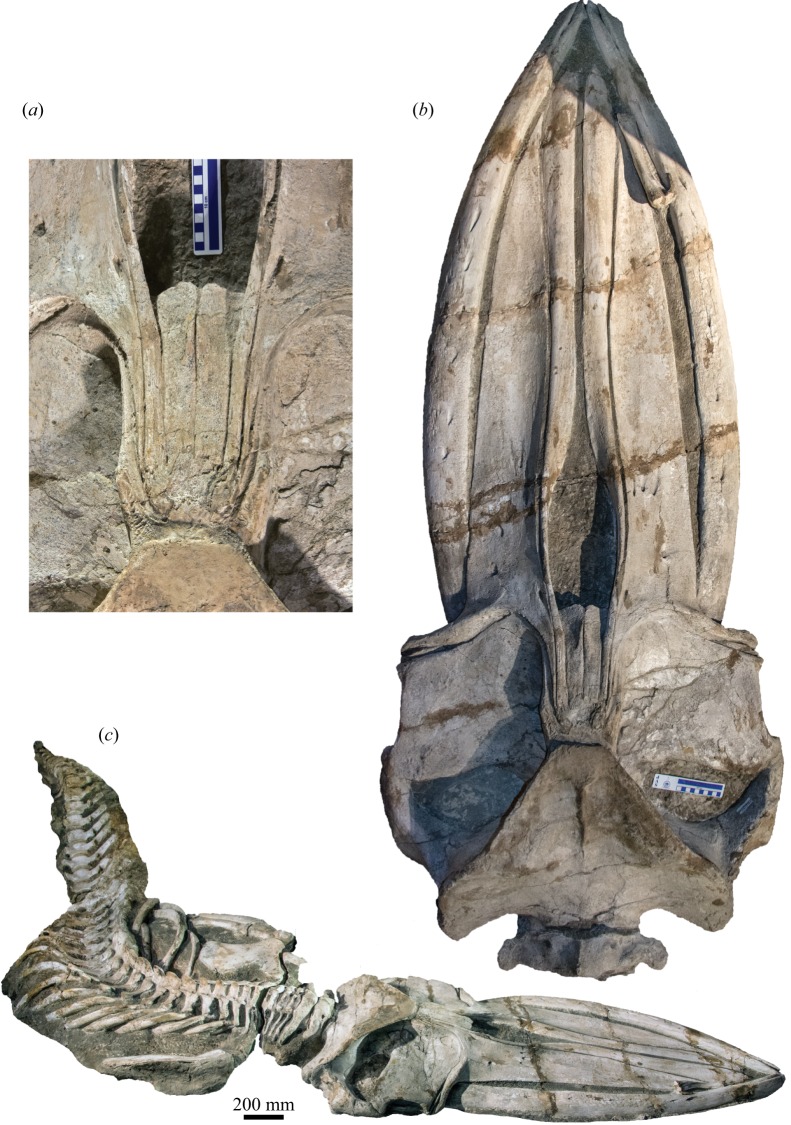
Paratype (KPM NNV730) of *Incakujira anillodefuego*. (*a*) Skull vertex and (*b*) skull, both in dorsal view; (*c*) skeleton as exhibited at the Kanagawa Prefectural Museum of Natural History, Odawara, Japan, in slightly anterior dorsolateral view. Dark area near tip of rostrum is a shadow caused by strong exhibition spotlights.

The paratype (KPM NNV730), which was also preserved in a dorsal position, is even more complete than the holotype and preserves virtually the entire vertebral column intact in articulation (figure 3). However, it is also somewhat less mature (see below) and less prepared, with the entire ventral surface of the specimen currently being inaccessible. Except for the scapula, the entire right flipper is buried beneath the chest, and it is currently unclear how much of it is preserved. Dorsoventral compression has damaged the medial portions of both supraorbitals and the anterior portions of both parietals. In addition, there is significant damage to the posterior portion of the vertex, the anterodorsal border of the left scapula and the anterior lumbar vertebrae, with L3 and L4 being nearly entirely destroyed. The transverse processes of all lumbar and caudal vertebrae are either severely damaged or missing. The pelves, 1–3 of the chevron bones and, possibly, some of the posterior-most caudal vertebrae are missing. The presence of the hyoid bones and/or mineralized baleen is uncertain.

The vertebral epiphyses of the holotype are firmly attached to their respective bodies in the anterior thoracic, posterior lumbar and caudal regions of the vertebral column, but only loosely connected or entirely dissociated elsewhere. Similarly, the proximal epiphyses of the humeri are largely ankylosed to the humeral shafts, whereas the distal epiphyses remain free. The holotype individual was, therefore, likely a subadult. In the paratype, the junctions between the vertebral epiphyses and their bodies are visible even in the posterior caudal region, and the proximal epiphysis of the left humerus, though attached to the shaft, remains clearly distinct. This individual, therefore, was likely somewhat younger than the holotype, which may explain its somewhat smaller size (total body length of 7.15 versus 8.25 m). The following description is based on holotype, unless indicated. Where they occur, differences with the paratype are made clear.

### 4.2. Skull

#### 4.2.1. Premaxilla

**Figure 4.**
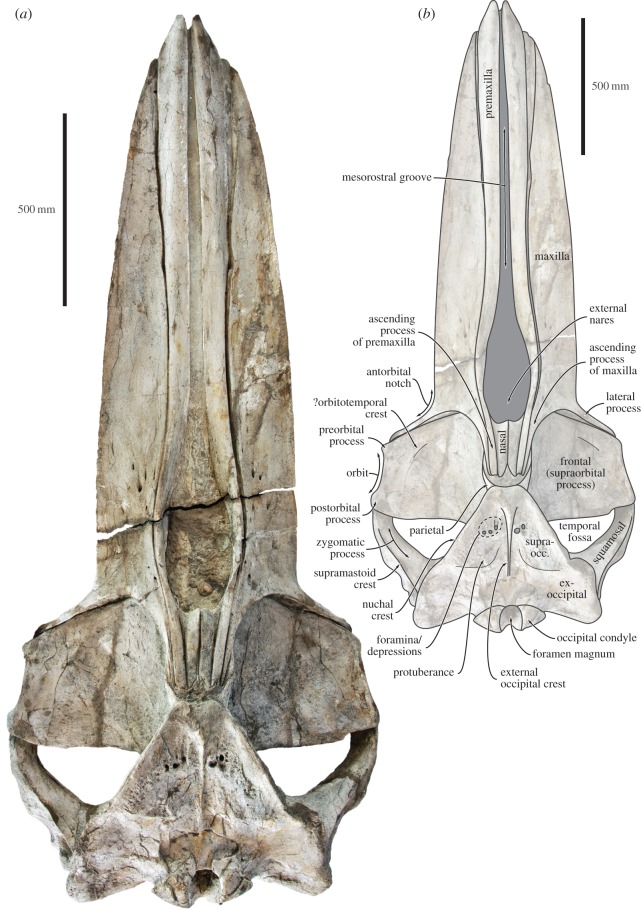
Holotype (GNHM Fs-098-12) cranium of *Incakujira anillodefuego*. (*a*) Photograph and (*b*) line drawing, both in dorsal view. supraocc., supraoccipital.

**Figure 5.**
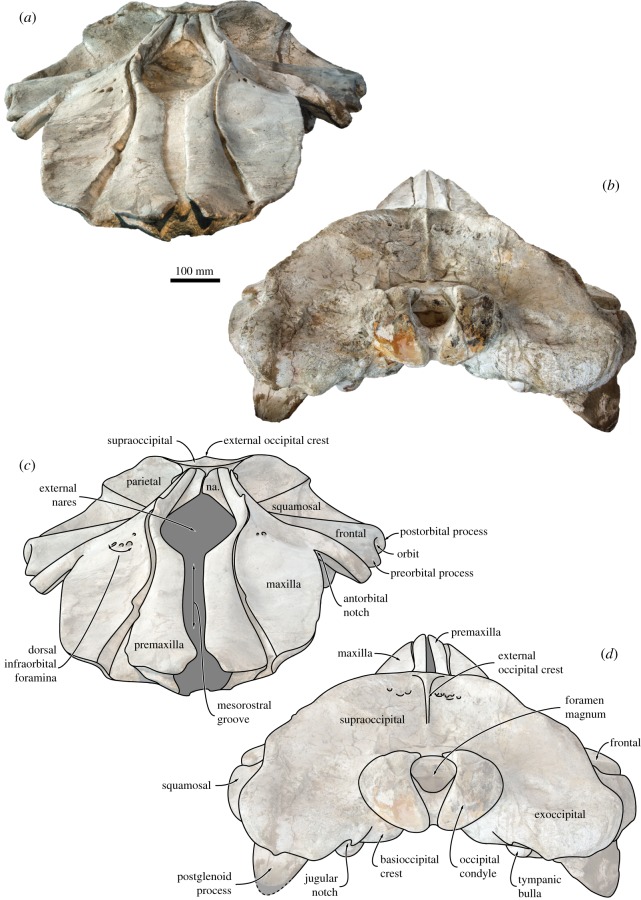
Holotype (GNHM Fs-098-12) cranium of *Incakujira anillodefuego*. (*a*,*b*) Photograph and (*c*,*d*) line drawings; (*a*,*c*) in anterior and (*b*,*d*) in posterior view.

**Figure 6.**
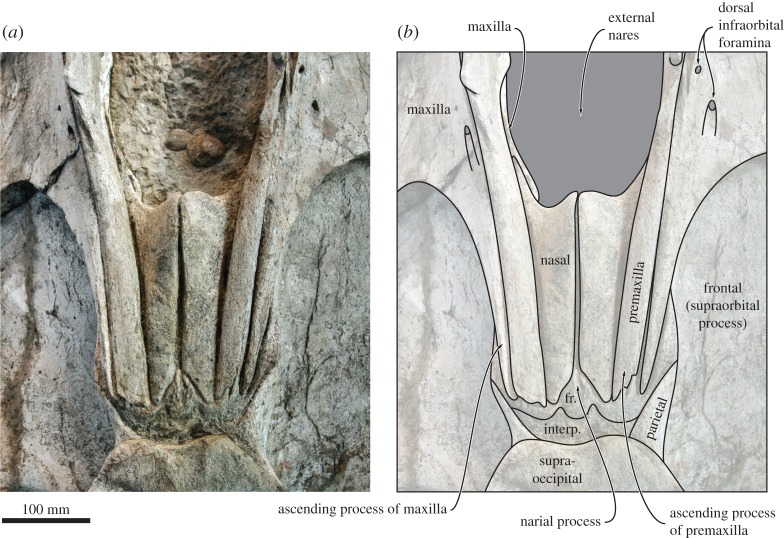
Skull vertex of the holotype (GNHM Fs-098-12) of *Incakujira anillodefuego*. (*a*) Photograph and (*b*) line drawing, both in dorsal view. fr., frontal; interp., interparietal.

**Figure 7.**
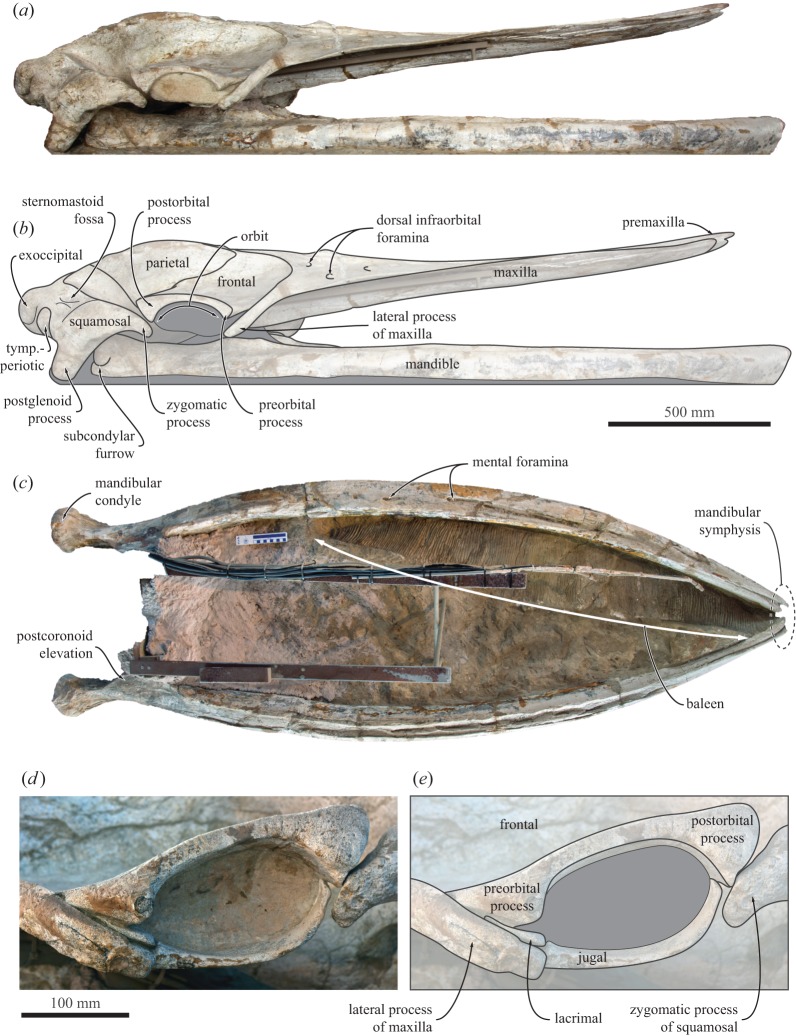
Holotype (GNHM Fs-098-12) cranium and mandibles of *Incakujira anillodefuego*. (*a*) Photograph and (*b*) line drawing of the skull in lateral view; (*c*) mandibles and fossilized baleen, in dorsal view; (*d*) photograph and (*e*) line drawing of the left orbit. tymp.-periotic, tympanoperiotic.

In dorsal view, the premaxilla is elongate and expanded anteriorly (figures 4 and 5). Anterior to the narial fossa, the premaxilla is flattened and abuts the maxilla along a smooth facet allowing a certain degree of transverse movement (figure 5). As preserved, the premaxillae never touch each other, not even at their apices. Just anterior to the level of the antorbital notch, the premaxilla becomes narrow and curves laterally to accommodate the wide narial fossa. The ascending process of the premaxilla is robust and parallel-sided all the way to its posterior tip, which is located roughly in line with, or just posterior to, the centre of the orbit (figures 4 and 6). In lateral view, the premaxilla barely rises above the level of the maxilla, expect for a distinct triangular eminence located near the point where the transverse distance between the premaxillae is at its widest (figure 7*a*,*b*).

#### 4.2.2. Maxilla

In dorsal view, the lateral margin of the maxilla is slightly convex (figure 4). There are several dorsal antorbital foramina, most of which are located at the level of anterior border of the narial fossa and end in short, anteriorly directed sulci. One each maxilla, the posterior-most dorsal infraorbital foramen opens into a sulcus rising on to the base of the ascending process (figure 6). Given its position and orientation, this foramen is likely homologous to the primary dorsal infraorbital foramen (*sensu* [42]) previously described in cetotheriids. The lateral process is elongated and oriented posterolaterally, thus forming a somewhat obtuse angle with the rostral margin. Anteriorly, the lateral process bears a faint, ridge-like antorbital process, which defines the dorsal margin of a smooth, widely open antorbital notch. Posterodorsally, the lateral process bears a crest that initially parallels the antorbital process, but then turns posteriorly and becomes confluent with the lateral border of the ascending process of the maxilla. As it turns, this crest overrides the anteromedial corner of the supraorbital process of the frontal and forms a small but distinct balaenopterid ‘pocket’.

The ascending process of the maxilla is narrow and somewhat triangular. For most of its length, it is markedly narrower than the adjacent ascending process of the premaxilla, but both terminate posteriorly at roughly the same level (figure 6). In lateral view, the maxilla forms a broad ventral keel running along almost the entire rostrum (figure 7). In both the holotype and the paratype, the lateral margin of the anterior portion of the maxilla is flattened, but originally would likely have gently curved ventrally to follow the outline of the (partially rotated) mandible, as in extant balaenopterids.

**Figure 8.**
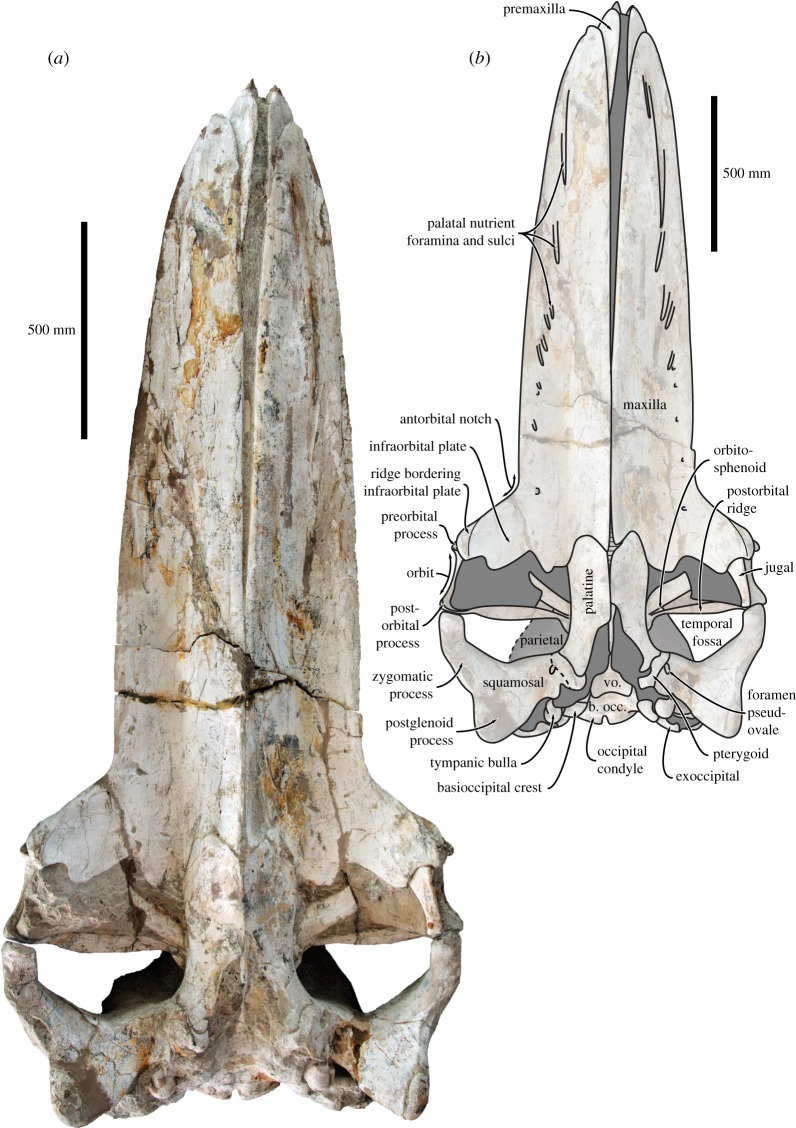
Holotype (GNHM Fs-098-12) cranium of *Incakujira anillodefuego*. (*a*) Photograph and (*b*) line drawing, both in ventral view.

In ventral view, the maxillae seemingly contact each other along the posterior half of the rostrum, but slightly diverge anteriorly to expose the ventral-most portion of the vomer. The extent of this divergence, as well as its absence further posteriorly, may partially be due to the post-mortem distortion of the rostrum and/or rostral kinesis. The palatal surface of each maxilla bears two sets of sulci and foramina: a medial one consisting of anteroposteriorly arranged sulci transmitting the greater palatine artery; and a lateral one consisting of a series of radially arranged sulci near the lateral margin of the rostrum, which originally housed branches of the superior alveolar artery supplying the baleen racks [46] (figure 8). Posteriorly, the maxilla forms a V-shaped suture with the palatine and gives rise to a well-developed infraorbital plate with a clearly defined embayment for the jugal. Anterolateral to the infraorbital plate, a well-developed ridge runs from the centre of the lateral process to a point ventral to the preorbital process of the frontal. Posterior to this ridge, the lateral-most portion of the infraorbital plate is markedly concave (figure 8).

#### 4.2.3. Nasal

In dorsal view, the nasal is robust, somewhat elongate and very slightly tapers posteriorly (figure 6). Its anterior border is deeply concave and located just posterior to the level of the anterior-most point of the supraorbital process. The anteromedial corner of the nasal is elevated into a well-developed sagittal crest, whereas the anterolateral corner is elongated and lodged in a small pocket formed by the maxilla. The posterior extremities of the nasals are separated by the narial process of the frontal, but still terminate approximately in line with the ascending processes of the maxilla and premaxilla.

#### 4.2.4. Palatine

In ventral view, the palatine is elongate and extends from the level of the preorbital process past the posterior border of the temporal fossa (figure 8). Posterolaterally, the palatine overrides the anterior portion of pterygoid. Posteromedially, the choanal margin of the palatine is broadly concave.

#### 4.2.5. Vomer

The vomer is largely obscured from view by the surrounding bones and sediment, but can be surmised to floor the mesorostral groove, as in other mysticetes. In ventral view, the rostral portion of the vomer is likely exposed between the medial margins of the maxillae for at least some of its length. Further posteriorly, the vomer emerges from underneath the palatines and forms a pronounced, broadly exposed vomerine crest (figure 8). Dorsal to this crest, the vomer forms a broad plate underlying the synchondrosis between the basioccipital and the basisphenoid.

#### 4.2.6. Lacrimal and jugal

The lacrimal is small, slightly elongate and wedged in its usual position between the lateral process of the maxilla and the preorbital process of the frontal (figure 7*d*,*e*). The jugal is effectively preserved *in situ*, except for a slight medial displacement that has caused its posterior end to become detached from the zygomatic process of the squamosal. In lateral view, the jugal is flattened and gently rounded. In ventral view, it is broad transversely and markedly expands anteriorly where it articulates with the infraorbital plate of the maxilla (figure 8).

#### 4.2.7. Frontal

In dorsal view, the frontal is clearly exposed on the vertex and form a well-developed, triangular narial process separating the nasals posteriorly (figure 6). The supraorbital process is broad anteroposteriorly, with its anteromedial corner extending anteriorly to the level of the antorbital notch (figure 4). The anterior border of the supraorbital process is straight or slightly concave, and oriented posterolaterally. The outline of the posterior border is more varied, being straight and oriented slightly anterolaterally in the holotype, but sinusoidal—owing to a more pronounced postorbital process—in the paratype (figures 3 and 4). The dorsal surface of the supraorbital process is flat and featureless, except for a shallow depression in its posterolateral corner and an anteriorly located eminence (only in the holotype) that may mark the position of the orbitotemporal crest (figure 4).

In anterior view, the supraorbital process is horizontal and abruptly depressed relative to the vertex (figure 5). In lateral view, both the preorbital and the postorbital processes are triangular, pointing ventrally (figure 7*d*,*e*). The posterior face of the postorbital process is flattened and approximates the tip of the zygomatic process of the squamosal, but over a relatively smaller surface than in extant *Balaenoptera*. The dorsal rim of the orbit is dorsoventrally thickened, more clearly so in the holotype (figure 7*d*,*e*). Medially, the frontal is overridden by the anterior portion of the parietal. In the holotype, the latter approximates the lateral border of the ascending process of the maxilla, thus almost separating the supraorbital process from the portion of the frontal exposed on the vertex (figure 6). In the paratype, the parietal and the maxilla are separated by a broad, anteroposteriorly oriented groove. Poor preservation makes it impossible to tell whether this groove is formed by the frontal, the parietal or both, but frontal seems to be the most likely option. In ventral view, the pre- and postorbital ridges are anteroposteriorly thickened, with the posterior border of the optic canal and orbit being located well anterior to the posterior margin of the supraorbital (figure 8).

#### 4.2.8. Parietal and interparietal

In dorsal view, the parietal is almost entirely obscured by the nuchal crest overhanging the temporal fossa, except for a small triangle that forms the lateral border of the vertex (figures 4 and 6). Between these triangles, the area separating the frontal anteriorly from the supraoccipital posteriorly appears to expose a distinct interparietal (this portion of the vertex is damaged in the paratype). In lateral view, the parietal broadly overrides much of the medial portion of the frontal, including the posteromedial corner of the supraorbital process (figure 7*a*,*b*). Anteriorly, the parietal overlaps with the posterior extremities of the nasal, premaxilla and maxilla. Posteriorly, the parietal contributes to a largely featureless parieto-squamosal suture.

#### 4.2.9. Supraoccipital, exoccipital and basioccipital

As far as can be told, all of the occipital elements are ankylosed in both the holotype and the paratype. In dorsal view, the supraoccipital forms an anteriorly pointing, narrowly truncated triangle with moderately sinusoidal lateral borders (figure 4). The apex of this triangle (damaged in the paratype) is slightly elevated and gives rise to a well-developed external occipital crest that terminates somewhat anterodorsal to the foramen magnum. The crest is located inside a longitudinal median trough, which is narrower and better defined in the holotype. Lateral to this trough, much of the centre of the supraoccipital consists of a somewhat elevated, triangular protuberance. Between this protuberance and the apex of the supraoccipital, the holotype shows several large, circular depressions or foramina, some of which open into short, anteriorly directed sulci (figure 4).

The nuchal crest forms the elevated lateral border of the supraoccipital and projects laterally over the inner wall of the temporal fossa. Posteriorly, the supraoccipital is fused to the wide exoccipital, which gives rise to a prominent occipital condyle situated on a distinct neck (figure 5). In the holotype, the occipital condyle projects posteriorly just beyond the level of the paroccipital process. The same is likely true of the paratype, but the articulation of the skull with the atlas prevents any direct observations in this case. The paroccipital process is dorsoventrally short, but anteroposteriorly thickened and thus prominent in both dorsal and lateral view. In ventral view, the basioccipital gives rise to a strong basioccipital crest, which is separated from the paroccipital process by the deep, dorsomedially oriented jugular notch.

#### 4.2.10. Squamosal

In dorsal view, the squamosal defines most of the posterior outline of the wide temporal fossa. The zygomatic process is robust yet elongate; its apex is oriented anteriorly, whereas its posterior portion projects anterolaterally—more distinctly so in the holotype (figures 3, 4 and 8). Posteriorly, the zygomatic process bears a well-developed supramastoid crest, which is further enhanced by the presence of a deeply excavated sternomastoid fossa (figure 7*a*). Somewhat posterior to the base of the zygomatic process, the supramastoid and nuchal crests converge anterior to the level of the occipital condyles. The squamosal fossa is short anteroposteriorly. There is a squamosal cleft, but no squamosal crease. The lateral border of the zygomatic process is offset from the lateral border of the exoccipital, but the resulting angle is less well defined than in other rorquals owing to the twisting of the postglenoid process (see below).

**Figure 9.**
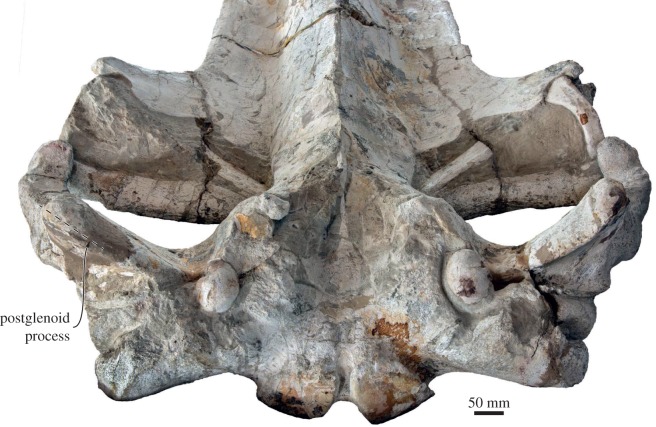
Holotype (GNHM Fs-098-12) cranium of *Incakujira anillodefuego.* Photograph in posterior view showing the twisted postglenoid processes of the squamosal.

In lateral view, the zygomatic process is dorsoventrally robust up to the level of the postorbital process. At this point, the apex of the zygomatic process becomes dorsoventrally flattened and abruptly curves ventrally (figure 7*a*,*b*). Ventral to the posterior portion of the supramastoid crest, the deep supramastoid fossa is divided in half by a horizontal ridge. The postglenoid process is well developed, parallel-sided, oriented slightly posteroventrally and descends well below the ventral margin of the exoccipital. In ventral view, the postglenoid process is weakly trapezoidal, with a poorly developed angle offsetting its ventral border from the posterior margin of the tympanosquamosal recess. The transverse axis of the postglenoid process is twisted clockwise on the left and anticlockwise on the right. The twisting is marked and appears largely symmetrical, and thus is unlikely to be the result of post-mortem distortion (figure 9). Posterior to the postglenoid process, the external acoustic meatus is broad anteroposteriorly, with its tall posterior border descending along the anterior face of the compound posterior process of the tympanoperiotic. Anteromedially, the squamosal gives rise to a robust falciform process that contributes most of the medial border of the foramen pseudovale and anteroventrally descends below the level of the anterior process of the periotic (figure 8).

#### 4.2.11. Alisphenoid and orbitosphenoid

All of the sphenoid bones are externally covered by the surrounding bones, except for a narrow portion of the orbitosphenoid exposed along the optic canal (figure 8) and a small triangular exposure of the alisphenoid in the temporal fossa, between the parietal, squamosal and pterygoid.

#### 4.2.12. Pterygoid

In ventral view, the pterygoid is exposed as a moderately thin strip between the palatine and the squamosal (figure 8). Posteriorly, the pterygoid forms about one quarter of the rim of the foramen pseudovale, with the remainder being contributed by the squamosal. Laterally, the pterygoid crosses from the basicranium into the temporal fossa, where it slightly expands and contacts the alisphenoid and the parietal. Medially, the pterygoid gives rise to a moderately developed hamular process. The latter is broken but, based on the shape of its base, can be surmised to have been finger-like. Dorsally, the pterygoid forms the walls of the pterygoid sinus fossa, with the medial lamina posteriorly contacting the basioccipital crest and the lateral lamina broadly overlapping the anterior process of the periotic. Most of the pterygoid sinus fossa remains obscured by matrix.

#### 4.2.13. Periotic

**Figure 10.**
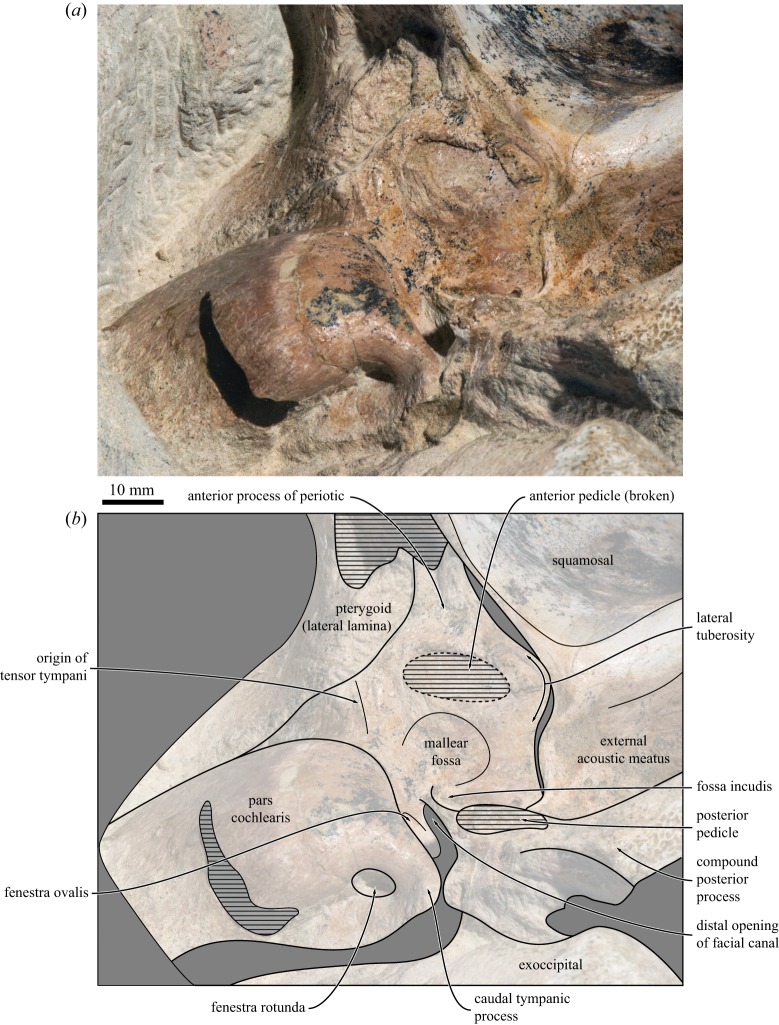
Left periotic of the holotype (GNHM Fs-098-12) of *Incakujira anillodefuego*. (*a*) Photograph and (*b*) line drawing, both in ventral view.

Only the partially prepared ventral portion of the left periotic of the holotype is accessible (figure 10). Most of the anterior process is obscured by the lateral lamina of the pterygoid. Anteroventral to the pars cochlearis and dorsal to the anterior pedicle of the tympanic bulla, a faint ridge marks the attachment of the tensor tympani. The lateral tuberosity is blunt, broadly triangular and located slightly posteroventral to the fused anterior pedicle of the tympanic bulla. The mallear fossa is shallow and extremely poorly defined. The pars cochlearis is cranially elongated, with its width greatly exceeding its length. There is no sign of a promontorial groove, although the latter could still occur near the currently inaccessible internal acoustic meatus. The fenestra rotunda is rounded and clearly separated from the aperture of the cochlear aqueduct; in medial view, it is obscured by the posteriorly bulging posteromedial corner of the pars cochlearis.

The caudal tympanic process is triangular and oriented posteriorly. Laterally, it approaches, but does not contact, the crista parotica. The fenestra ovalis and distal opening of the facial canal remain partially covered by sediment, but appear to be clearly separated. The facial canal is partially floored by a thin shelf bearing the fossa incudis. The posterior process is anteroposteriorly compressed, but relatively thick compared with living balaenopterids. Externally, the posterior process is exposed on the lateral skull wall and appears to be mortised into the surrounding squamosal, somewhat resembling the condition in *Eschrichtius*.

#### 4.2.14. Tympanic bulla

**Figure 11.**
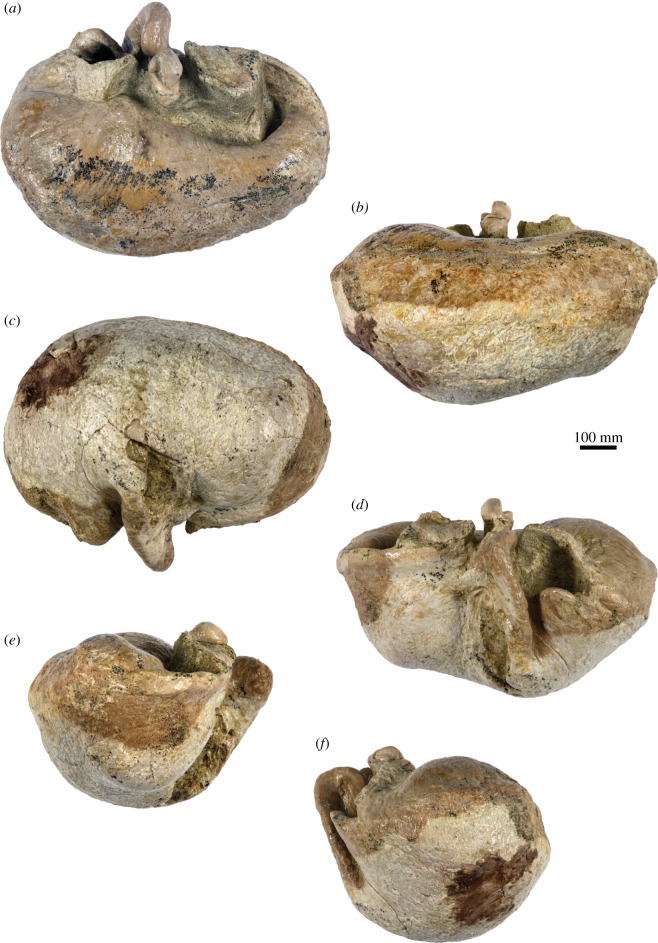

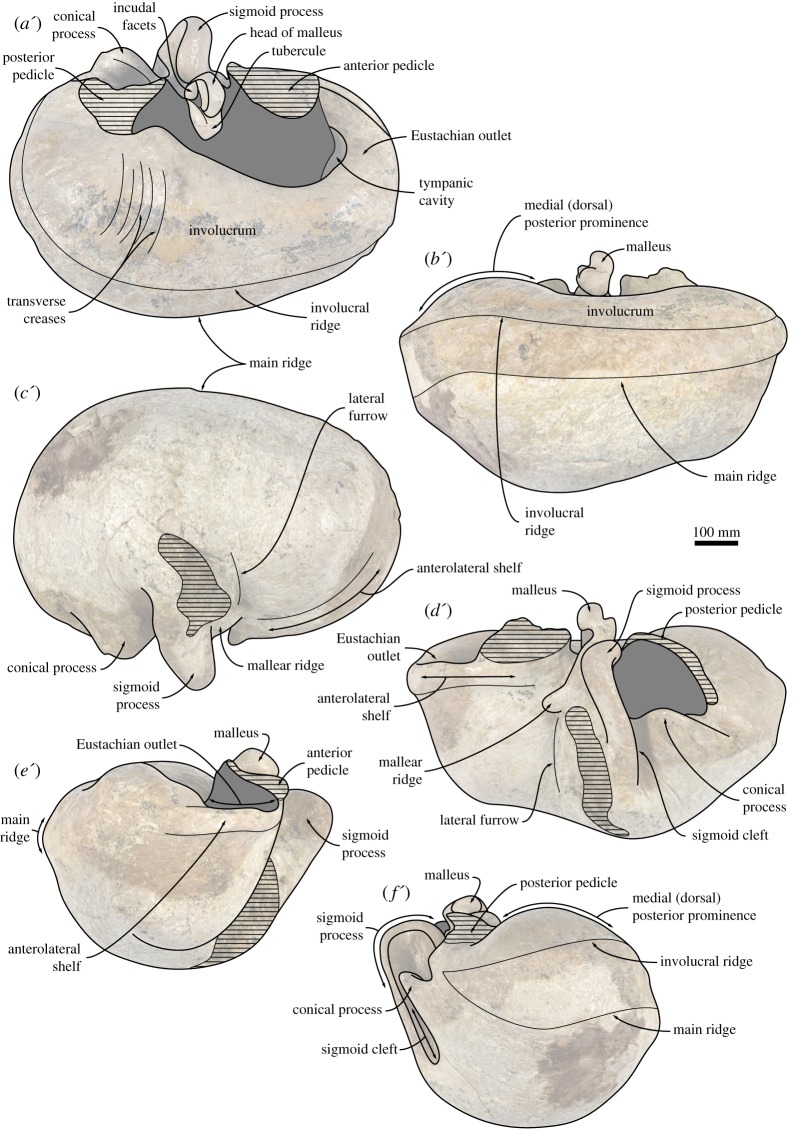
Tympanic bulla of the holotype (GNHM Fs-098-12) of *Incakujira anillodefuego*. (*a*) Dorsal, (*b*) medial, (*c*) ventral, (*d*) lateral, (*e*) anterior and (*f*) posterior view; (*a*–*f*) photographs and (*a*′–*f*′) line drawings.

In dorsal view, the tympanic bulla is roughly kidney-shaped (figure 11*c*). Anteriorly, the Eustachian outlet is defined by a thick (approx. 2 mm) bony crest that runs along the anterior-most portion of the outer lip and posteriorly joins the anterior pedicle. The shape of the latter is partially obscured by a portion of the periotic that has remained attached to the bulla, but appears to be robust and long anteroposteriorly (approx. 20.5 mm). The dorsal border of the sigmoid process is slightly twisted so that its dorsomedial corner points posteriorly. Posteriorly, the sigmoid process does not overhang the conical process, but the two structures are connected by a bony bridge that medially closes off the sigmoid cleft. The conical process is dorsally rounded and bent laterally. Medially, it carries the posterior portion of the tympanic sulcus, which is developed as a relatively sharp bony crest and posteriorly rises on to the posterior pedicle. The posterior pedicle is robust, but its exact shape is obscured by breakage. The involucrum is well developed along the entire length of the tympanic bulla, but markedly broader posteriorly than anteriorly; its dorsal surface bears some fine transverse sulci. The involucral ridge (= inner posterior prominence) is distinct along the entire length of the bone, but laterally retracted from the main ridge (= outer posterior prominence).

In lateral view (figure 11*d*), the mallear ridge is relatively poorly developed and located just ventral to the posterior-most portion of the anterior pedicle. Dorsally, the mallear ridge gives rise to the anterior process of the malleus, which carries a short but well-defined sulcus for the chorda tympani. Ventral to the mallear ridge there is a vertically oriented lateral furrow. The dorsal portion of the sigmoid process leans slightly anteriorly, reflecting the twisting of its dorsal margin. The sigmoid cleft, which defines the posterior border of the sigmoid process, is roughly vertical and does not turn anteriorly as in basal chaeomysticetes. The conical process is well developed and approximately parabolic in outline.

In ventral view (figure 11*a*), the surface of the tympanic bulla is convex transversely. Anteriorly, there is a shallow anterolateral shelf (*sensu* [47]). The anterolateral corner of the outer lip is mildly inflated and forms a distinct lobe that is posteriorly bounded by the lateral furrow. In medial view (figure 11*b*), the dorsal surface of the involucrum appears concave, thanks to a notable remnant of the inner (now dorsal) posterior prominence. The main and involucral ridges are roughly parallel and separated from each other by a band of roughened bone running along the entire medial margin of the bulla. There is no median furrow or interprominential notch. In anterior view (figure 11*e*), the rim of the Eustachian outlet is roughly horizontal and does not markedly descend below the level of the involucrum. The lateral margin of the sigmoid process is oriented somewhat dorsolaterally, resulting in the entire process appearing slightly deflected. In posterior view (figure 11*f*), the main and involucral ridges remain distinct and converge at the level of the posterior pedicle. There is no distinct interprominential ridge (*sensu* [48]).

#### 4.2.15. Malleus

**Figure 12.**
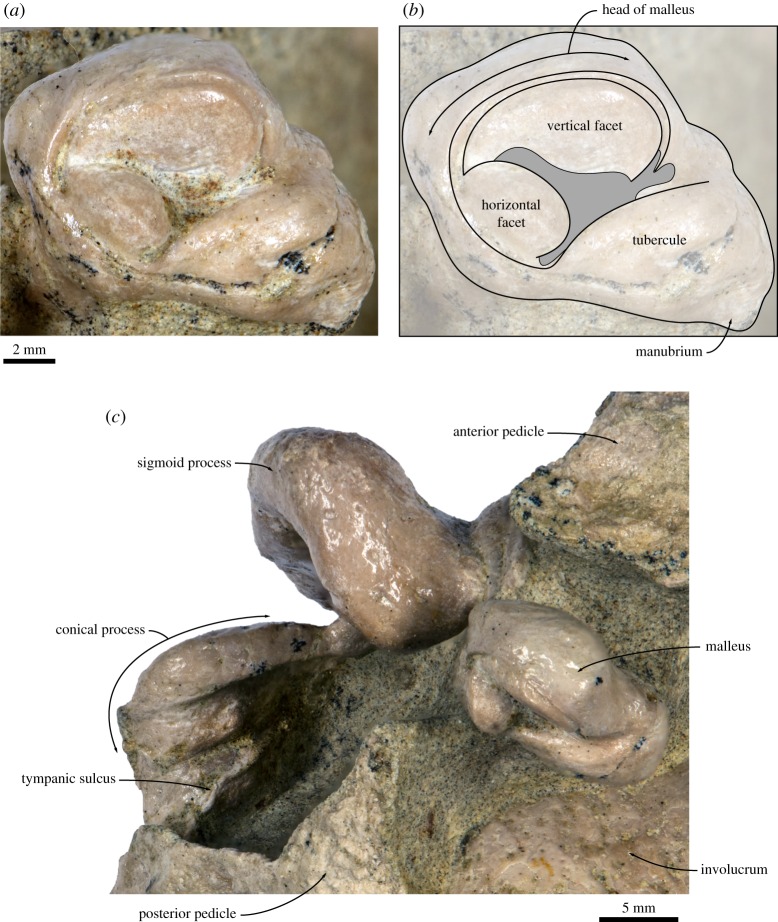
Malleus of the holotype (GNHM Fs-098-12) of *Incakujira anillodefuego*. (*a*) Photograph and (*b*) line drawing, both in posterior view; (*c*) position of malleus on the tympanic bulla, in slightly posterior dorsomedial view.

In anterior view, the anterior process of the malleus and sulcus for the chorda tympani rise obliquely from the mallear ridge past the dorsomedial corner of the sigmoid process, without fusing to the latter as in most extant balaenopterids (figure 12). The head of the malleus is rounded and ventrally excavated by the sulcus for the chorda tympani. The manubrium is only slightly smaller than the head, oriented approximately medially, and terminates in a ventrally pointing hook. The position of the muscular process is obscured by matrix. In posterior view, the head of the malleus carries the perpendicularly arranged incudal facets, with the vertical one being at least twice as large as its horizontal counterpart. Medially, the incudal facets are separated from the hook-like manubrium by a marked sulcus.

#### 4.2.16. Mandible

In dorsal view, the body of the mandible is moderately bowed laterally (figure 7*c*). The mandibular symphysis is unsutured. The anterior-most portion of the mandible is oriented nearly vertically and not noticeably twisted. The mandibular neck is slightly recurved, thus giving the mandible as a whole a somewhat sigmoidal outline. The postcoronoid elevation is well developed and projects well beyond the medial border of the mandibular neck. In medial view, the mandibular body roughly maintains a constant height anteroposteriorly. The mandibular foramen is dorsoventrally short (approx. half the height of the body) and located entirely posterior to the level of the coronoid process. The condyle is slightly raised above the dorsal border of the neck and oriented posteriorly. Ventrally, the condyle is separated from the angular process by a deep subcondylar furrow, which continues around the posterior border of the mandible and is visible in lateral view (figure 7*a*,*b*). The angular process is massive and projects posteriorly slightly beyond the level of the condyle. The coronoid process is broken but appears to have been bent laterally. There is no satellite process.

#### 4.2.17. Baleen

**Figure 13.**
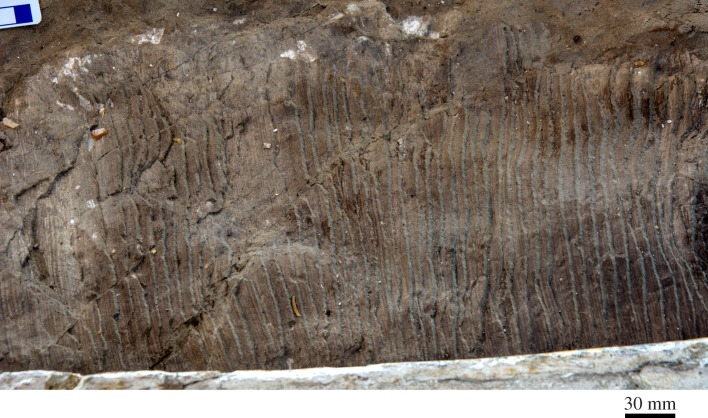
Fossilized baleen in the holotype (GNHM Fs-098-12) of *Incakujira anillodefuego*.

As in other material from the Pisco Formation [19,29], the holotype preserves traces of mineralized baleen. A large portion of the ?left rack, comprising more than 170 individual baleen plates, has become detached from the palate, and is preserved on the floor of the mouth along the inside of the left mandible (figures 7*c* and 13); there is no sign of its right counterpart. The average spacing of the individual plates, based on 20 measurements, is approximately 3.3 mm along the anterior portion of the rack, and 3.4 mm along the posterior portion, within the range of extant *Balaenoptera acutorostrata* [49]. Because the baleen plates themselves have decayed, plate density cannot be measured reliably. Nevertheless, assuming a plate thickness of 1.0–1.5 mm suggests an estimated plate density of just over 2 plates cm^−1^, similar to *B. acutorostrata*, and slightly lower than in *Caperea marginata* and *Balaenoptera borealis* [49]. Near the posterior edge of the rack, intraplate spacing increases to about 5–6 mm. A similar posterior increase occurs in *B. acutorostrata* [49], and may indicate that the rack of the holotype, though detached, retains its original anteroposterior orientation.

### 4.3. Postcrania

#### 4.3.1. Forelimb

**Figure 14.**
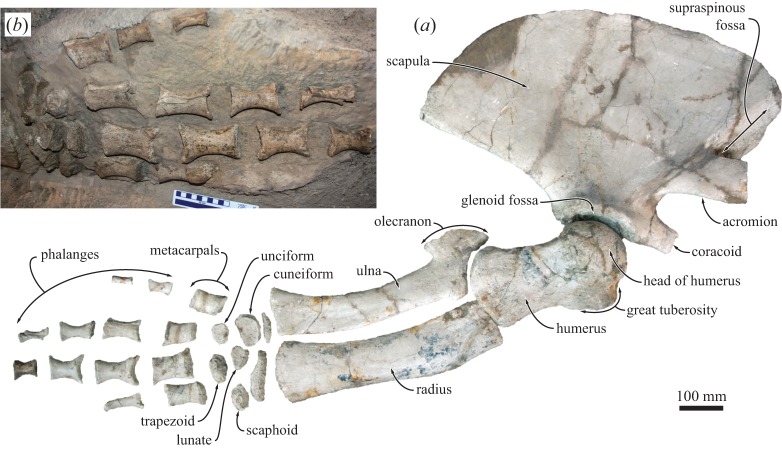
Forelimb of the holotype (GNHM Fs-098-12) of *Incakujira anillodefuego*. (*a*) Right forelimb and (*b*) left manus. Note that most of the phalanges in (*a*) are reconstructed based on their left counterparts.

The forelimb overall resembles that of extant rorquals (figure 14). The scapula is anteroposteriorly elongate, with a narrow supraspinous fossa, a well-developed, broad acromion and a robust coracoid process. The humerus is shorter than the forearm (radius and ulna) and relatively straight in lateral view, with the head being only slightly angled posteriorly. Both the proximal and distal epiphysis are wider than the shaft. The great tuberosity is well developed and distally confluent with what appears to be a noticeable deltoid tuberosity. The radial and ulnar facets are oriented at a shallow angle to each other and similar in size. In lateral view, the radius is slightly more robust than the ulna and terminates in an anteroposteriorly flared distal epiphysis. Close to the proximal epiphysis, the anterior border of the radius slightly bulges anteriorly, in the position previously interpreted as the insertion of the brachialis muscle in basilosaurids [50]. In extant cetaceans, the brachialis appears to be absent [51]. The ulna bears a well-developed olecranon process that rises proximally to the level of the distal humeral epiphysis. Like that of the radius, the distal epiphysis of the ulna is anteroposteriorly flared. The manus is tetradactyl, and contains five rounded and, presumably, only partially ossified carpals [52].

#### 4.3.2. Cervical vertebrae

**Figure 15.**
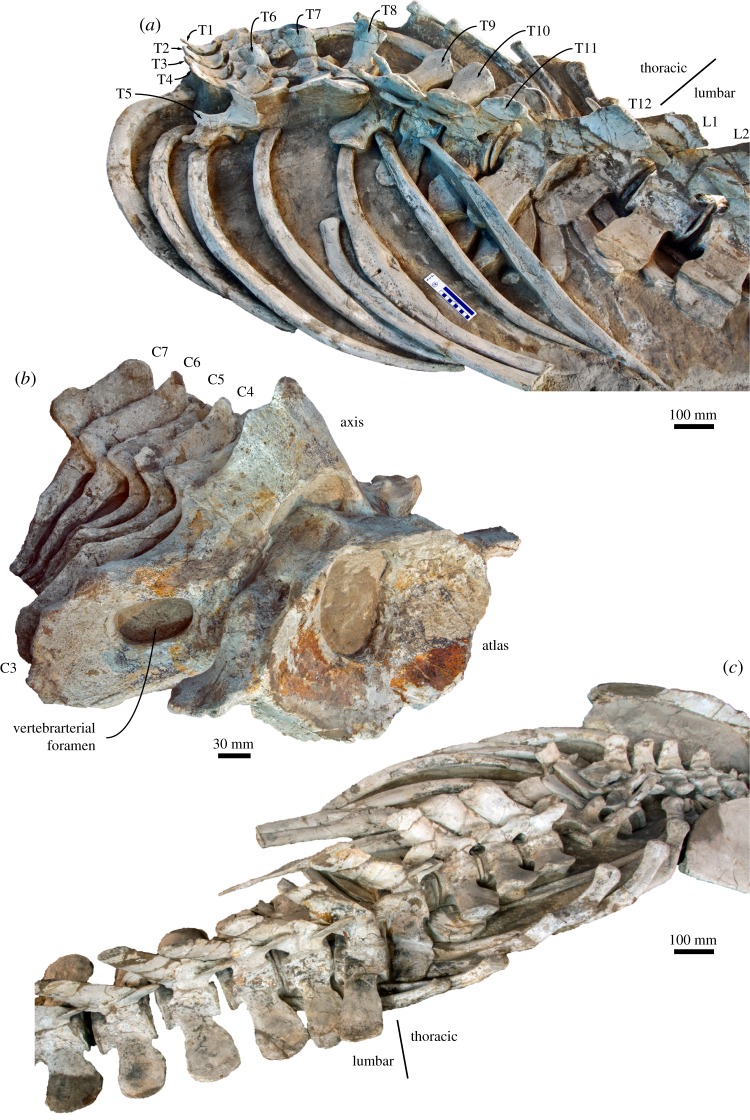
Vertebrae and ribs of the holotype (GNHM Fs-098-12) of *Incakujira anillodefuego*. Thoracic and anterior lumbar vertebrae in (*a*) left and (*c*) right dorsolateral view; (*b*) cervical vertebrae in oblique anterolateral view.

The seven cervical vertebrae are unfused. In anterior view, the atlas has a broadly concave articular surface for the occipital condyle and a single, dorsoventrally flattened transverse process (figure 15*b*). The neural arch barely rises above the level of the articular facet. In lateral view, a large foramen for the suboccipital nerve perforates the side of the neural arch and opens into a broad, anterolaterally directed sulcus. The axis is the largest of the cervicals and characterized by a tall neural arch and well-developed, distally fused di- and parapophyses enclosing a moderately sized vertebrarterial foramen (figure 15*b*). Posteriorly, the axis bears a clearly defined postzygapophysis. C3–C7 are similar in size and morphology, with anteroposteriorly broad neural arches, well-developed pre- and postzygapophyses, and elongate diapophyses. Details of the vertebral bodies and the parapophyses are obscured by matrix in both specimens, but at least C3 appears to have a well-developed lower transverse process that is laterally fused to its upper counterpart.

#### 4.3.3. Thoracic vertebrae

The holotype has 12 vertebrae with transverse articular facets and corresponding ribs (figure 15), whereas the paratype has 13. In anterior view, the vertebral bodies are wider than high. Distinct metapophyses occur from T7 onwards in both specimens. Initially (T7–T11), the metapophyses are robust, with a rounded outline in lateral view, and project anteriorly far beyond the anterior border of the body (figure 15). Further posteriorly, the metapophyses become more proximodistally elongate and oriented anterodorsally. In lateral view, the distal portions of the spinous processes are anteroposteriorly expanded and squared. In dorsal view, the posterior throacics bear a centrally located projection on the anterior border of the transverse process.

#### 4.3.4. Lumbar vertebrae

The number of lumbar vertebrae in the holotype is unclear owing to the partial loss of the posterior portion of the vertebral column. In the paratype, incomplete preparation and breakage of the transverse processes obscures the position of the last lumbar; however, there are at least 9, based on the position of a bone fragment that may be a remnant of the first chevron bone, and no more than 11, based on the position of the first confidently identified chevron bone. In anterior view, the vertebral bodies of the lumbars are oval or round. The transverse processes are oriented horizontally. The metapophyses are oriented dorsally and broad anteroposteriorly, but overall shorter than on the thoracic vertebrae. In dorsal view, the transverse processes are rounded and broad anteroposteriorly.

#### 4.3.5. Caudal vertebrae

The paratype preserves 16–18 caudal vertebrae, depending on which vertebra is identified as Ca1. The posterior-most three vertebrae are preserved within a separate block of matrix, and it is unclear whether any additional vertebrae were lost as a result of this breakage. If so, however, their number would have been small. Dorsoventral foramina piercing the base of the transverse process occur from Ca3/Ca5 onwards. A residual spinous process occurs up until Ca6/Ca8, and the neural canal remains distinct up until Ca7/Ca9. Posterior to Ca6/Ca8, the dorsoventral foramina perforate the vertebral body itself; from Ca9/Ca11 onwards, they become markedly larger and are connected dorsally by a well-developed transverse sulcus.

#### 4.3.6. Chevron bones

The paratype preserves remnants of at least six chevron bones. Of these, the second and third are the largest and most complex in lateral view, with rounded ventral keels that are markedly longer anteroposteriorly than their articulations with the vertebrae. Further posteriorly, the chevron bones become smaller and simplified, appearing approximately round in lateral view.

## 5. Discussion

### 5.1. Intraspecific variation

The holotype and paratype resemble each other most clearly in the morphology of their vertices, *viz*. (i) the extremely narrow ascending process of the maxilla; (ii) the broadly exposed frontal giving rise to a well-developed narial process; (iii) the broad, elongate nasal bearing an anterior sagittal crest; and (iv) the triangular, narrowly truncated supraoccipital shield. Other similarities include (v) the presence of a well-developed external occipital crest bordered by a longitudinal trough and triangular protuberances, and (vi) the prominent paroccipital process. By contrast, the two specimens differ in the number of ribs (12 in the holotype, 13 in the paratype) and the shape of the posterior border of the supraorbital process, which is straight in the holotype, but distinctly sinusoidal in the paratype. In addition, the holotype is somewhat larger and has a slightly better-developed occipital condyle. In light of the somewhat younger ontogenetic age of the paratype and variation in the number of thoracic vertebrae among extant balaenopterids [53], we do not consider these differences sufficient to warrant the description of separate taxa, and instead attribute them to intraspecific variation.

### 5.2. Phylogeny

Our total evidence analysis reveals *I. anillodefuego* to be a rorqual. Balaenopterid phylogeny is vexed by marked contradictions between molecular and morphological analyses, as well as often inconsistent results across different morphological studies. Morphological data generally support a monophyletic Balaenopteridae to the exclusion of grey whales (Eschrichtiinae, following [15,54]), as well as a monophyletic extant *Balaenoptera* to the exclusion of *M. novaeangliae* [55–58]. Specifically, characters potentially supporting *Balaenoptera* include a posteriorly widening, squared ascending process of the maxilla (shared with *Diunatans*); fusion of the anterior process of the malleus to the dorsomedial corner of the sigmoid process of the bulla; an elongate, triangular postorbital process broadly abutting the zygomatic process of the squamosal; and the presence of a squamosal crease. Nevertheless, molecular and total evidence analyses frequently nest both *Eschrichtius* and *Balaenoptera* inside extant *Balaenoptera* [13–15,59] and, as a result, have even called for the break-up of the genus [16].

**Figure 16.**
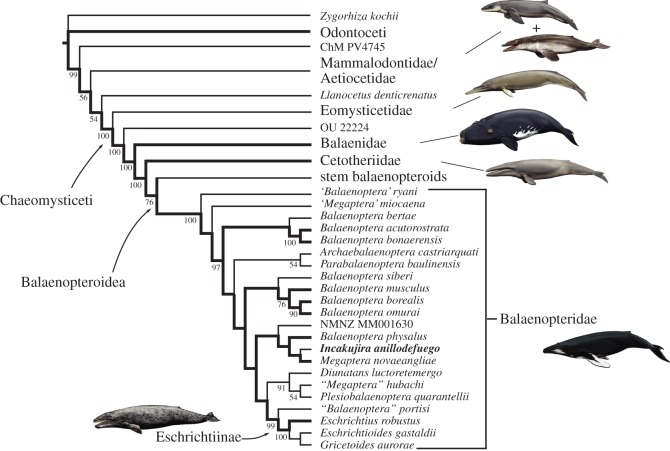
Phylogenetic relationships of *Incakujira anillodefuego*. Consensus tree showing all compatible clades (‘allcompat’ option in MrBayes) summarizing the results of the total evidence analysis. Only posterior probabilities greater than 50% are shown. Bold branches mark extant lineages. Drawings of cetaceans by C. Buell.

*Incakujira anillodefuego* bears all of the major balaenopterid hallmarks, including an abruptly depressed supraorbital process of the frontal, an elongate ascending process of the maxilla that anteroposteriorly overlaps with the parietal and forms a ‘pocket’ with the underlying supraorbital process, a cranially elongated pars cochlearis, an anterolateral shelf on the tympanic bulla, a mandible with a sinusoidal dorsal outline, a dorsoventrally narrow mandibular foramen, and a subcondylar furrow that wraps around the posterior face of the mandible and is visible in lateral view [56,60]. In line with these observations, the results of our phylogenetic analysis place *Incakujira anillodefuego* inside crown Balaenopteridae, as sister to the extant humpback whale *M. novaeangliae* (figure 16; electronic supplementary material, figure S1). Specifically, *I. anillodefuego* and *M. novaeangliae* share the presence of (i) a relatively narrow premaxilla (relative to the maxilla) halfway along the rostrum (Char. 5); (ii) a narial process of the frontal (Char. 77); (iii) an elongate external occipital crest (Char. 116); (iv) a triangular, posteriorly oriented caudal tympanic process, contrasting with the ventrally bulging caudal tympanic process of their common sister taxon, NMNZ MM001630 (Char. 166); and (v) a crest-like tympanic sulcus. Nevertheless, this relationship is poorly supported, as are most other clades within the family as a whole (figure 16).

The morphology-only analysis also recovers *I. anillodefuego* inside crown Balaenopteridae, but this time as sister to a monophyletic *Balaenoptera* excluding both *Megaptera* and *Eschrichtius* (electronic supplementary material, figure S2). As in previous studies [15,59], the position of extant balaenopterids in our total evidence tree thus appears to be determined largely by molecular evidence, rather than morphology. This phenomenon is not problematic *per se*, as the molecular data are likely to improve the overall phylogenetic accuracy of our results [61]. Nevertheless, it also highlights a marked difference in the molecular and morphological signals likely caused by an abundance of convergent, and thus potentially misleading, traits. In light of such contradictory evidence, the relationships of *Incakujira* remain uncertain. One way of tackling this issue from a morphological perspective will require detailed studies into whether and how features seemingly diagnostic of *Balaenoptera* correlate with ecology, and thus hopefully a better understanding of their ecomorphological plasticity.

### 5.3. Feeding ecology

Despite recent advances in understanding the biomechanics of rorqual lunge feeding [7–12], morphological correlates of this behaviour that could potentially be detected in fossils remain poorly understood. Most of the anatomical features clearly and uniquely associated with lunge feeding, such as an expandable throat pouch, a non-synovial (fibrous) craniomandibular joint and a symphyseal organ sensing jaw motion and throat pouch inflation [9], consist of soft tissue currently not known to leave clear osteological traces. A laterally bowed mandible bearing an outwardly deflected coronoid process has previously been interpreted as a potential indicator of gulping behaviour [48,62,63], but also occurs in taxa currently interpreted as skim- or suction feeders, such as eomysticetids (e.g. laterally bent coronoid process in *Yamatocetus canaliculatus*, KMNH VP000017) and cetotheriids (e.g. *P. nana*, MNHN SAS1618). A more taxonomically restricted trait present exclusively in balaenopterids, including *I. anillodefuego*, is the presence of a broad, abruptly depressed supraorbital process with a thickened, posteriorly rounded postorbital ridge that may act as a pulley for the well-developed temporalis muscle [7,64]. In agreement with another recent study on rorqual evolution [55], we interpret the presence of this morphology as a potential indicator of lunge feeding, and thus suggest that *Incakujira* likely also employed this strategy.

Nevertheless, the feeding apparatus of *Incakujira* differs from that of living rorquals in one major regard. In the extant species, a fibrous craniomandibular joint allows the mandible to rotate in three directions, namely longitudinally (alpha rotation), dorsoventrally (delta rotation) and laterally (omega rotation) [7]. Omega rotation is partially made possible by a transversely oriented postglenoid process, which, thanks to its orientation, presents no obstacle to sideways movements of the condyle. In *Incakujira*, however, the postglenoid process is markedly twisted (figure 9), and thus presumably constrained or even prevented omega rotation. The effect or function of this feature in the context of lunge feeding is uncertain, as restricted omega rotation would limit expansion of the oral cavity, and thus also the amount of water that can be engulfed.

It is possible that *Incakujira*, despite otherwise resembling other balaenopterids, had at least partially moved away from lunge feeding in favour of an alternative feeding strategy. An analogous example for such a process may be provided by the extant sei whale, *B. borealis*, whose rostral morphology, finely fringed baleen and (compared with other extant balaenopteroids) less expandable throat pouch appear to be somewhat convergent on skim-feeding right whales [65]. Restricted omega rotation and the relatively high baleen plate density of *Incakujira* are both consistent with facultative skim feeding, and imply that this species may have targeted relatively small-sized prey, such as copepods. Interestingly, small prey size—as inferred from high plate density and thin baleen bristles—has also been hypothesized for another, largely undescribed balaenopterid from a more northern exposure of the Pisco Formation at Cerro Colorado [66]. However, there is currently no information on whether this fossil may be conspecific with, or closely related to, *I. anillodefuego*. Additional data on the morphology of the Cerro Colorado material and/or dedicated microstructural analysis of the baleen preserved with GNHM Fs-098-12 may help to clarify the feeding strategy of *I. anillodefuego* in the future.

## 6. Conclusion

*Incakujira anillodefuego* is a new genus and species of Late Miocene rorqual based on two exceptionally preserved specimens from the Pisco Formation of Peru. Phylogenetically, *Incakujira* is close to, and possibly nested within, crown Balaenopteridae, but ongoing contradictions between morphological and molecular analyses mean that the evolutionary relationships of the family as a whole remain unsettled. Overall, *I. anillodefuego* closely resembles extant balaenopterids and may have employed a similar lunge-feeding strategy. However, it differs from all extant taxa in having a twisted postglenoid process of the squamosal, which likely reduced its lunge-feeding capability and suggests that *Incakujira* may have been able to pursue other, additional feeding strategies, such as skimming.

## Supplementary Material

Figure S1. Phylogenetic relationships of Incakujira gamagoriensis. Majority-rule consensus tree showing all compatible clades (“allcompat” option in MrBayes) showing the full results of the total evidence analysis. Only posterior probabilities >50% are shown.

## Supplementary Material

Figure S2. Phylogenetic relationships of Incakujira gamagoriensis. Majority-rule consensus tree showing all compatible clades (“allcompat” option in MrBayes), based on the results of the analysis using morphological data only. Only posterior probabilities >50% are shown.

## Data accessibility

The new, illustrated morphological codings and the full matrix are available from MorphoBank, project 2452 (full matrix stored in the ‘Documents’ section). This published work and the nomenclatural acts it contains have been registered in ZooBank. The LSID for this publication is: urn:lsid:zoobank.org:pub:DF696255-5BD3-435C-B3C3-EE39F9EB33F3.
