# Supplementary figures and images for: A new Miocene baleen whale from the Peruvian desert
Source: R Soc Open Sci. 2016 Oct 5;3(10):160542. doi: 10.1098/rsos.160542 (PMC5098998; doi:10.1098/rsos.160542)

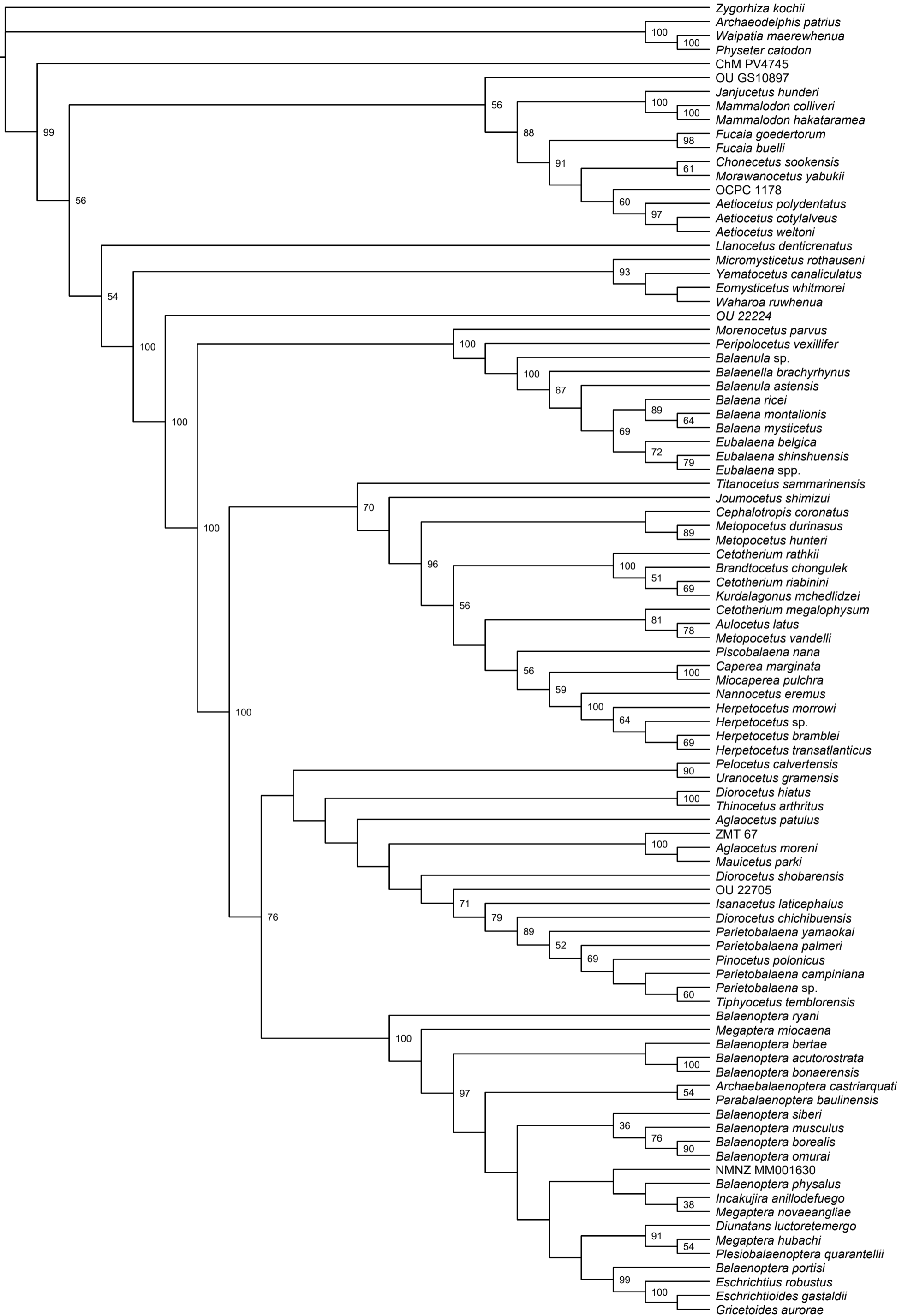

Supplement: Figure S1. Phylogenetic relationships of Incakujira gamagoriensis. Majority-rule consensus tree showing all compatible clades (“allcompat” option in MrBayes) showing the full results of the total evidence analysis. Only posterior probabilities >50% are shown. [file rsos160542supp1.pdf]

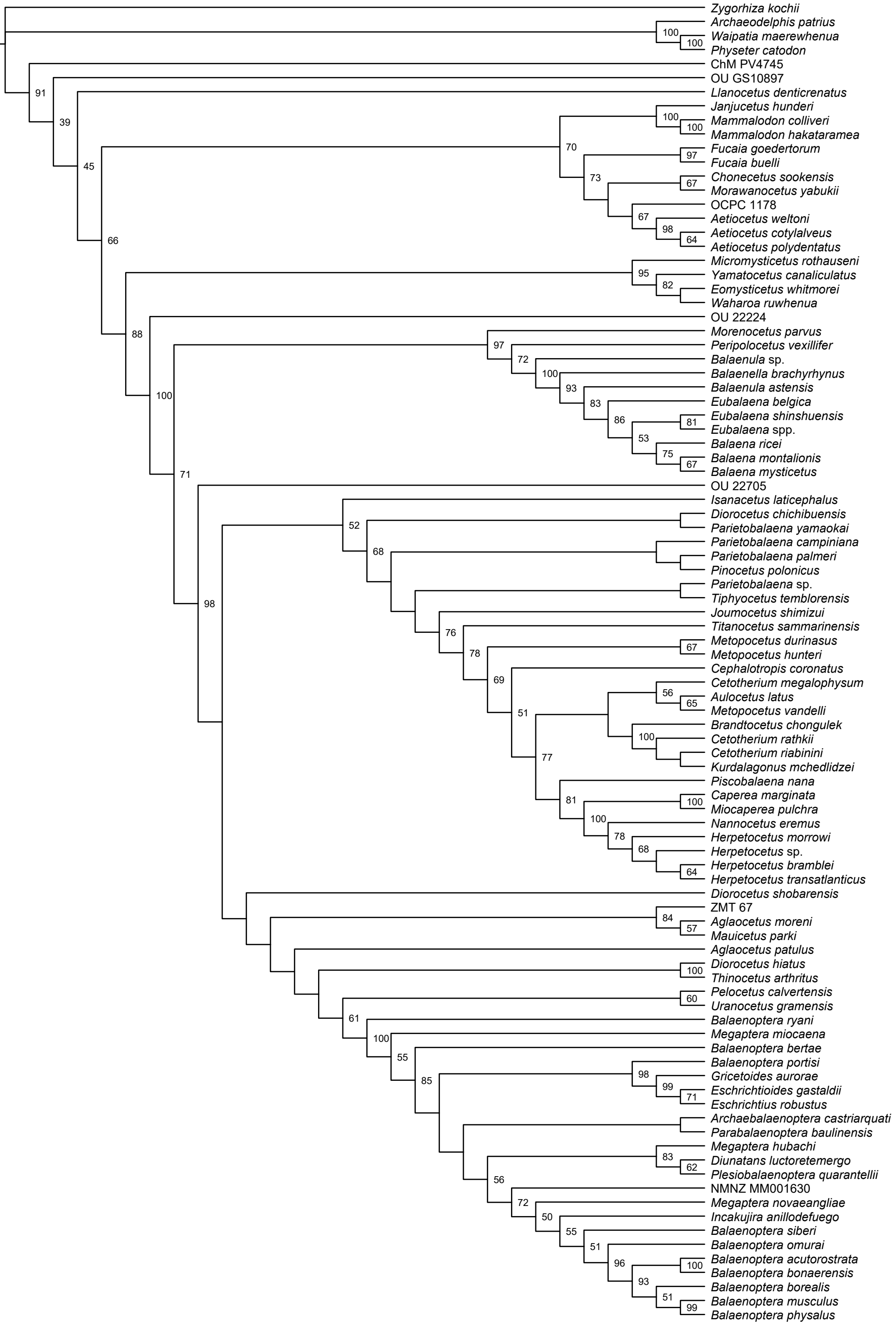

Supplement: Figure S2. Phylogenetic relationships of Incakujira gamagoriensis. Majority-rule consensus tree showing all compatible clades (“allcompat” option in MrBayes), based on the results of the analysis using morphological data only. Only posterior probabilities >50% are shown. [file rsos160542supp2.pdf]
